# Salt Tolerance Improvement in Rice through Efficient SNP Marker-Assisted Selection Coupled with Speed-Breeding

**DOI:** 10.3390/ijms20102585

**Published:** 2019-05-26

**Authors:** Md Masud Rana, Takeshi Takamatsu, Marouane Baslam, Kentaro Kaneko, Kimiko Itoh, Naoki Harada, Toshie Sugiyama, Takayuki Ohnishi, Tetsu Kinoshita, Hiroki Takagi, Toshiaki Mitsui

**Affiliations:** 1 Department of Life and Food Sciences, Graduate School of Science and Technology, Niigata University, Niigata 950-2181, Japan; f16m502g@mail.cc.niigata-u.ac.jp (M.M.R.); takamatsutakeshi@yahoo.co.jp (T.T.); k-neko@gs.niigata-u.ac.jp (K.K.); kimi@agr.niigata-u.ac.jp (K.I.); naharada@agr.niigata-u.ac.jp (N.H.); sugiyama@agr.niigata-u.ac.jp (T.S.); 2 Agronomy Division, Bangladesh Rice Research Institute, Gazipur-1701, Bangladesh; 3 Faculty of Agriculture, Niigata University, Niigata 950-2181, Japan; mbaslam@gs.niigata-u.ac.jp; 4 Center for Education and Research of Community Collaboration, Utsunomiya University, Utsunomiya 321-8505, Japan; ohnishi@cc.utsunomiya-u.ac.jp; 5 Kihara Institute for Biological Research, Yokohama City University, Yokohama 244-0813, Japan; tkinoshi@yokohama-cu.ac.jp; 6 Faculty of Bioresources and Environmental Sciences, Ishikawa Prefectural University, Ishikawa 921-8836, Japan; h-takagi@ishikawa-pu.ac.jp

**Keywords:** *hst1*, Na^+^ accumulation, SNP, rapid generation advance, salt tolerant, variant annotation, whole-genome sequencing

## Abstract

Salinity critically limits rice metabolism, growth, and productivity worldwide. Improvement of the salt resistance of locally grown high-yielding cultivars is a slow process. The objective of this study was to develop a new salt-tolerant rice germplasm using speed-breeding. Here, we precisely introgressed the *hst1* gene, transferring salinity tolerance from “Kaijin” into high-yielding “Yukinko-mai” (WT) rice through single nucleotide polymorphism (SNP) marker-assisted selection. Using a biotron speed-breeding technique, we developed a BC_3_F_3_ population, named “YNU31-2-4”, in six generations and 17 months. High-resolution genotyping by whole-genome sequencing revealed that the BC_3_F_2_ genome had 93.5% similarity to the WT and fixed only 2.7% of donor parent alleles. Functional annotation of BC_3_F_2_ variants along with field assessment data indicated that “YNU31-2-4” plants carrying the *hst1* gene had similar agronomic traits to the WT under normal growth condition. “YNU31-2-4” seedlings subjected to salt stress (125 mM NaCl) had a significantly higher survival rate and increased shoot and root biomasses than the WT. At the tissue level, quantitative and electron probe microanalyzer studies indicated that “YNU31-2-4” seedlings avoided Na^+^ accumulation in shoots under salt stress. The “YNU31-2-4” plants showed an improved phenotype with significantly higher net CO_2_ assimilation and lower yield decline than WT under salt stress at the reproductive stage. “YNU31-2-4” is a potential candidate for a new rice cultivar that is highly tolerant to salt stress at the seedling and reproductive stages, and which might maintain yields under a changing global climate.

## 1. Introduction

Projected climate change will aggravate a variety of abiotic stresses of rice plants, including salinity, heat, drought, and submergence, thus reducing world rice production [1,2,3,4]. At the same time, we must increase global rice production by at least 70% to feed the anticipated 9.6 × 10^9^ people by 2050 [5,6]. Under these conditions, the improvement of the salinity tolerance of locally grown high-yielding rice cultivars is one of the most promising breeding objectives by which to meet global food demand.

Rice is considered the most salt-sensitive cereal crop [7], with a threshold EC_e_ (electrical conductivity of saturated extract) of 3 dSm^−1^, above which yield starts to decline [8,9,10]. Salinity imposes osmotic effects, ion toxicity, and nutritional imbalance and substantially affects almost all phases of growth [7,11]. Possible salt tolerance mechanisms in rice involve ion homeostasis and compartmentalization, ion transport and uptake, biosynthesis and accumulation of osmoprotectants, osmolytes, and compatible solutes, activation of antioxidant enzymes for ROS detoxification, and hormone modulation [12,13,14,15,16,17]. The *Saltol* [18,19] and *SHOOT K^+^ CONCENTRATION 1* [20,21] genes have been identified from major quantitative trait loci (QTLs) of salt-tolerant landraces Pakkali and Nona Bokra, respectively. These QTLs have been introgressed into some widely grown, high-yielding rice cultivars to improve salt tolerance [22,23,24,25,26], but the rate of improvement is slow.

Rice biotechnology has made advances in identifying single nucleotide polymorphisms (SNPs) controlling salinity tolerance [27,28,29,30,31]. Mutant lines of “Hitomebore” were generated by treatment with ethyl methanesulfonate (EMS), an inducer of nucleotide substitutions, and isolated a salt-tolerant line carrying the *hitomebore salt tolerant 1* (*hst1*) gene. The causative SNP conferring the high salinity tolerance of the *hst1* mutant line corresponded to the third exon of the *Os06g0183100* gene, which is predicted to encode a B-type response regulator designated *OsRR22*. We backcrossed the *hst1* line with “Hitomebore” to breed the salt-tolerant cultivar “Kaijin”, with a yield ability of 5.88 t ha^−1^ [32,33]. “Yukinko-mai” is an early-maturing standard cultivar derived from a cross between “Yukino-sei” and “Domannaka” at the Niigata Agricultural Research Institute’s Crop Research Center; it has a high yield potential of 6.84 t ha^−1^ [34] and is tolerant to high temperatures during grain filling [35].

To combat earthquake- and tsunami-induced soil salinity in Japan, it is crucial to improve the salt resistance of locally grown popular rice cultivars, most of which are salt sensitive [32,36,37,38]. In addition, developing Japanese cultivars for international appeal and fine-tuning their yield performance under various ecosystems around the world are time-demanding tasks. To generate new rice cultivars quickly, in response to evolving consumer preferences and crises, we crossed the salt-tolerant “Kaijin” with “Yukinko-mai” to develop a salt-tolerant line with elite agronomic traits through the use of marker-assisted selection (MAS). MAS is the most advanced tool yet developed for the precise introgression of genes of interest into elite rice cultivars [39,40], and allows breeders to recover most of the recurrent parent genome in only two or three generations [41]. Salt, heat, and drought stress-responsive genes or QTLs revealed by recent advances in genomics and biotechnology are being used for MAS of rice all over the world [42,43,44].

In recent years, rapid generation-advance technology called “speed-breeding” has been used to shorten the generation cycle, accelerating the progress of genomics and breeding studies in multiple crops [45,46,47,48]. This technique has been used for the genetical improvement of rice, such as recombinant inbred lines, backcrossed inbred lines, and isogenic cultivars [46,47]. The speed breeding method has been reported for six major crops such as spring wheat (*Triticum aestivum*), durum wheat (*Triticum durum*), barley (*Hordeum vulgare*), chickpea (*Cicer arietinum*), pea (*Pisum sativum*), and canola (*Brassica napus*), that uses a prolonged photoperiod to reduce the generation time [48]. Nagatoshi and Fujita [49] developed a breeding technique for short-day soybean plant applying supplemental CO_2_ in combination with long-day and appropriate temperature cycles. Using speed-breeding, which combines temperature, light duration, and humidity control, tiller removal, and embryo rescue, breeders can obtain four to five advanced generations in a year [45,46].

In this study, we precisely transferred the *hst1* (*OsRR22*) gene, which confers salinity tolerance, from “Kaijin” into high-yielding “Yukinko-mai” (WT) rice through SNP MAS coupled with speed-breeding. We sequenced the whole genome of a BC_3_F_2_ *hst1* homozygous line and determined the genome recovery rate. We also examined important physiological and biochemical parameters of the BC_3_F_3_ generation that confer salt tolerance and evaluated the phenotype under salt stress and normal field conditions.

## 2. Results

### 2.1. Breeding Scheme for Development of Advanced Plant Material

**Figure 1:**
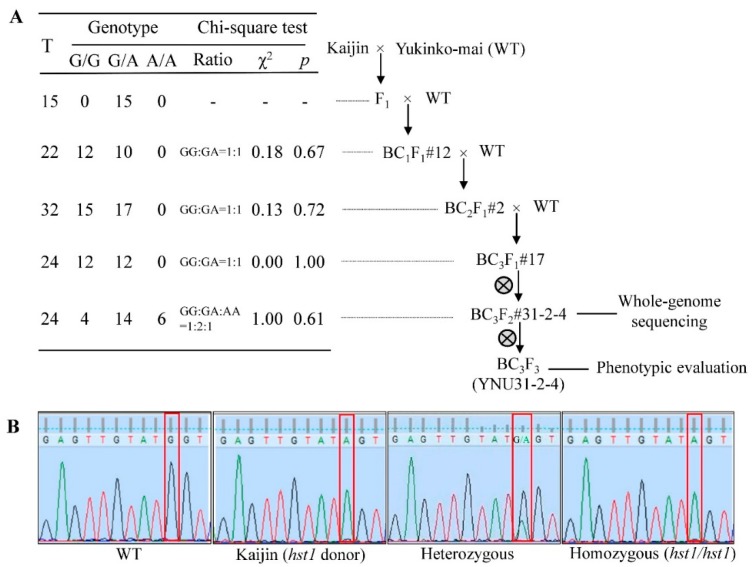
Single nucleotide polymorphism (SNP) marker-aided introgression of *hst1* from “Kaijin” into “Yukinko-mai”. (**A**) The *hst1* gene was transferred from highly salt-tolerant “Kaijin” into “Yukinko-mai”. “Kaijin” was backcrossed to “Yukinko-mai” (WT) 3 times followed by 2 rounds of self-pollination. The table shows the selection results at each generation: T, total number of tested plants; G/G, number of plants not carrying donor allele; G/A, number of plants carrying donor allele in heterozygous state; A/A, plants carrying donor allele in homozygous state. The number after “#” is the individual plant number used in backcrossing or self-pollination. (**B**) Advanced breeding individuals were genotyped by direct sequencing. The red box represents nucleotide 1975 of the *OsRR22* locus, which is responsible for salt tolerance.

We introgressed the salt-tolerance *hst1* gene from “Kaijin” into the genetic background of the high-yielding “Yukinko-mai” by three backcrosses followed by two rounds of self-fertilization (Figure 1A). To accelerate the breeding cycle, we used a biotron speed-breeding system, with controlled temperature and daylength, restriction of tillers, and embryo rescue (Figure S1). At each cross, the plants produced a good quantity of fertilized seed; the cross success rate ranged between 54% and 69% (Table S1), and seeds from three or four plants were sufficient to develop new progeny. Each advanced generation took approximately 70 days from germination to flowering and 10 days from pollination to embryo rescue (Figure S1). The total duration of each generation varied according to days to flowering. Using this speed-breeding technique, we developed the BC_3_F_3_ population, carrying our desired allele in the homozygous state, in six generations and 17 months.

### 2.2. Introgression of hst1 into “Yukinko-mai” (WT) and Genotyping of Advanced Progeny Using SNP Marker

We used SNP-based genotyping by Sanger sequencing to identify plants harboring the donor allele in each breeding round. A SNP in the *OsRR22* (*hst1*) gene confers the salinity tolerance of the donor parent “Kaijin”. The breeding lines were selected on the basis of target peaks of G/A heterozygosity (nucleotide 1975 of the *OsRR22* locus) in the F_1_, BC_1_F_1_, BC_2_F_1_, and BC_3_F_1_ generations and of A/A homozygosity at the same locus in the BC_3_F_2_ generation (Figure 1B). In the F_1_ to BC_3_F_1_ generations, we obtained two genotypes: either homozygous, lacking the donor allele (G/G), or heterozygous (G/A) (Figure 1A). The heterozygous BC_3_F_1_ population was self-pollinated to develop BC_3_F_2_ lines that carried the donor allele in the homozygous state (A/A). BC_3_F_2_ plants (A/A) morphologically similar to the recurrent parent were self-pollinated to develop the BC_3_F_3_ generation. We sequenced the whole genome of the BC_3_F_2_ line #31-2-4 to compare it with the parental genome, and characterized it.

### 2.3. Recovery Rate and Characterization of BC_3_F_2_ #31-2-4 Genome

**Figure 2:**
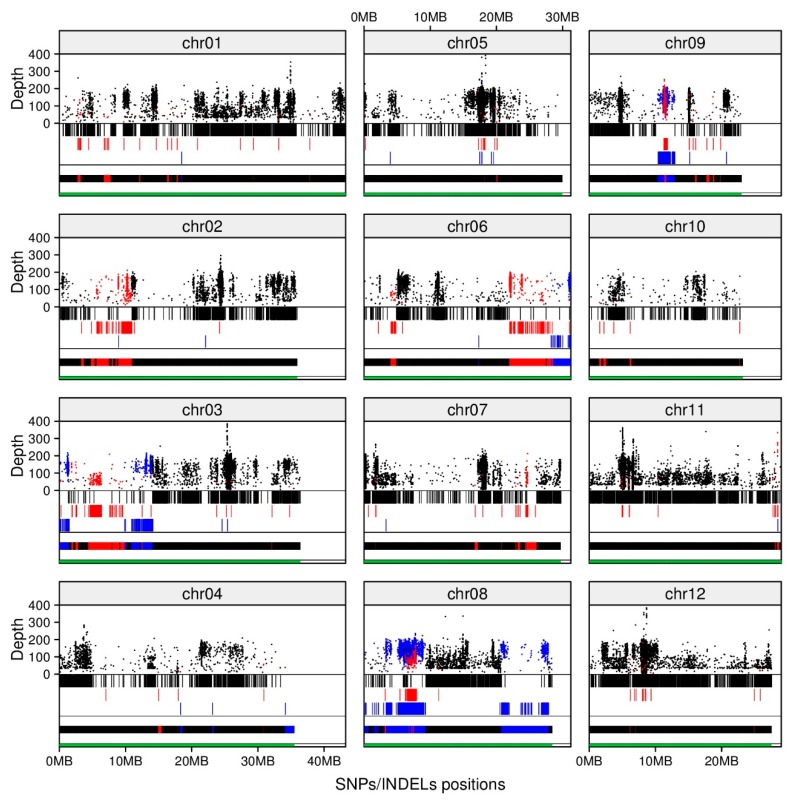
Positions and depths of SNPs/indels and genotype blocks on BC_3_F_2_ #31-2-4 genome. Dots show coverage depth of SNPs/indels. Vertical bars show their positions by genotype (color). Horizontal bar shows densities of SNPs/indels densities in 10,000-nt sliding window by genotype (colour). Black, “Yukinko-mai”-type homozygous; red, “Kaijin”-type homozygous; blue, heterozygous. Green bars represent chromosome length.

To investigate the genetic similarities between our advanced line and the parents, we analyzed BC_3_F_2_ #31-2-4 using whole-genome sequencing. After we filtered out low-reliability SNPs/indels, #31-2-4 showed 118 454 SNPs/indels, comprising 106 288 “Yukinko-mai”-type homozygous alleles, 3157 “Kaijin”-type homozygous alleles, and 9009 heterozygous alleles (Table 1). These SNPs/indels lie across the genome with deep coverage (Figure 2, dots), indicating high resolution and successful genome-wide genotyping. Allele types formed dense blocks on chromosomes (Figure 2, vertical bars), clearly showing recovered regions (“Yukinko-mai” homozygous blocks), “Kaijin” genome segments (“Kaijin” homozygous blocks), and unfixed segments (heterozygous blocks) (Figure 2, horizontal bar). “Yukinko-mai” chromosomes (Chrs.) 5, 11, and 12 were recovered almost completely. There were small “Kaijin” segments in Chrs. 1, 4, 7, 9, and 10, large “Kaijin” segments in Chrs. 2, 3, and 6, small heterozygous segments in Chr. 4, and large heterozygous segments in Chrs. 3, 6, 8, and 9. Interestingly, we identified some genotype blocks overlapping other genotype blocks (Figure 2, chr08, 5–10 Mb; chr09, 10–12 Mb), resulting from continuous recombination events in these extremely short regions [50,51]. We calculated the genome recovery rate from the number of “Yukinko-mai” alleles out of the total number; the BC_3_F_2_ genome recovered 93.5% of the “Yukinko-mai” genome, from 89.7% homozygous alleles and 7.6% heterozygous alleles (Table 1; Section 4.7). This score is close to the theoretical value of 93.7% following three backcrosses and one self-fertilization. In addition, 2.7% of the BC_3_F_2_ genome was “Kaijin” homozygous and 7.6% remained unfixed as heterozygous (Table 1).

**Figure 3:**
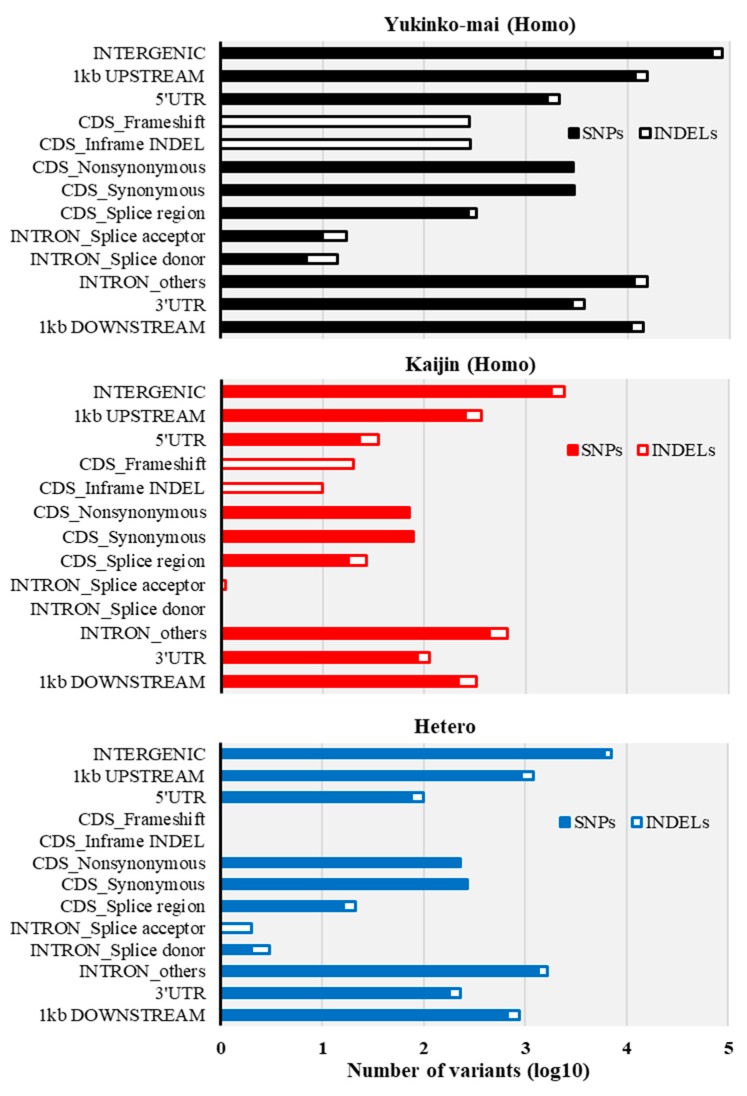
Sequence Ontology analysis of BC_3_F_2_ SNPs/indels. Sequence Ontology is based on SnpEff terms with minor modifications. Closed bars, SNPs; open bars, indels. Black, “Yukinko-mai”-type homozygous, red, “Kaijin”-type homozygous; blue, heterozygous.

To estimate the effects of variants on phenotypes, we listed variants causing protein sequence alterations (e.g., frameshift and in-frame indels, non-synonymous SNPs, SNPs/indels at splice donor/acceptor sites etc.) in SnpEff software for Sequence Ontology and effect prediction (Figure 3). In BC_3_F_2_, 207 “Kaijin” homozygous and 536 heterozygous variants were found in exon and splice sites. Only 71 and 230, respectively, of those caused protein sequence alterations (Figure 3; Table S2). These non-synonymous changes occurred in only four genes for agronomic traits (Table 2).

### 2.4. Field Evaluation of the “YNU31-2-4” for Main Agronomic Traits

We evaluated “YNU31-2-4” in the field to compare its major agronomic traits with those of the parents. We found a significant difference in days-to-heading between “YNU31-2-4” and WT: some plants headed significantly earlier than WT (Table 3; Figure S2B). Heading date was associated negatively with the *Os09g0356200* genotype and positively with the *hst1* genotype. These results support the accuracy of gene-based GWAS [53], and also suggest the potential early flowering function of *hst1* or the effect of unknown flowering-related genes. Grain width, grain thickness, and 1000-grain weight were significantly higher in “YNU31-2-4” plants than WT (Table 3). The *hst1* mutant also had a wider grain than WT [32]. Together these results, at least partly, suggest that *hst1* might be involved in the increase of grain width. In contrast, other important morphological traits of “YNU31-2-4” were highly similar to those of WT, particularly flag leaf color, length, and width, plant height, tiller number per plant, panicle number per plant, panicle length, spikelet number per panicle, grain yield per plant, aboveground biomass per plant, and grain length (Table 3; Figure S3A–C).

### 2.5. Salinity Tolerance of the “YNU31-2-4” Line at Seedling Stage

**Figure 4:**
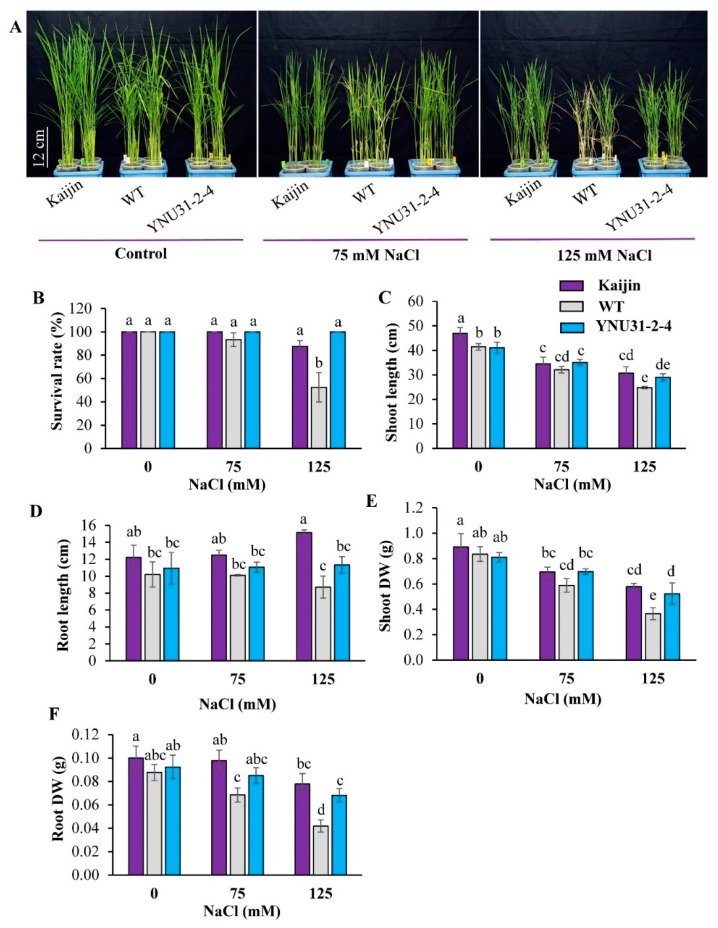
Salinity tolerance of BC_3_F_3_ line “YNU31-2-4” at seedling stage. (**A**) Phenotypic comparison of “Kaijin”, WT, and “YNU31-2-4” seedlings grown in 0, 75, or 125 mM NaCl for two weeks. (**B**) Survival rates, (**C**) shoot length, (**D**) root length, (**E**) shoot dry weight, and (**F**) root dry weight of seedlings shown in **A**. Data in **B**–**F** are mean ± SD of four independent biological replicates; data in **E** and **F** are dry weight (DW) of 10 plants in each treatment. Bars labeled with the same letter are not statistically different (Tukey’s test, *p* < 0.05).

We evaluated the salinity tolerance of “Kaijin”, “Yukinko-mai” (WT), and “YNU31-2-4” seedlings during three weeks at 0, 75, and 125 mM NaCl. At 0 mM NaCl, there was no phenotypic difference between “YNU31-2-4” and WT (Figure 4A). Under salt stress, however, the WT leaves were rolled, whereas those of “Kaijin” and “YNU31-2-4” remained flat and stayed green even at 125 mM NaCl. Further, 125 mM NaCl reduced the survival of WT seedlings to 52.5%, whereas all seedlings of “YNU31-2-4” survived (Figure 4B). Moreover, “Kaijin” and “YNU31-2-4” had better shoot and root growth under salinity vs. WT (Figure 4C–F); at 125 mM NaCl, “YNU31-2-4” had 30% and 38% better shoot and root dry weight, respectively than WT. In a separate experiment (Figure S4), at 0 mM NaCl, there were no significant differences in leaf relative water content or chlorophyll content between WT and “YNU31-2-4” plants. Under salt stress, the “YNU31-2-4” plants were able to maintain significantly higher relative water content and chlorophyll levels than WT (Figure S4A,B). Under the control condition, “YNU31-2-4” had significantly higher proline content than the parents. Exposure to salinity led to a considerable increase in proline levels in all genotypes, and to 1.6× the WT level in “YNU31-2-4” (Figure S4C). These results clearly indicate that “YNU31-2-4” has stronger salt tolerance than “Yukinko-mai” (WT) at the seedling stage.

### 2.6. Na^+^ and K^+^ Content in Shoot and Roots of the Tested Genotypes under Salt Stress

**Figure 5:**
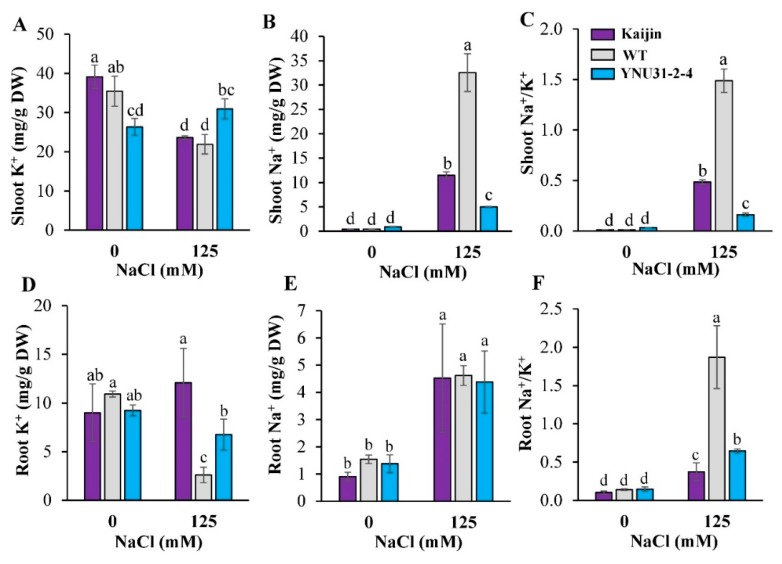
Na^+^ and K^+^ content in shoot and roots of the tested genotypes under salt stress. (**A**) Shoot K^+^, (**B**) shoot Na^+^, (**C**) shoot Na^+^/K^+^, (**D**) root K^+^, (**E**) root Na^+^, and (**F**) root Na^+^/K^+^ of 20-day-old seedlings. Data are mean ± SD of three independent biological replicates. Bars with the same letter are not statistically different (Duncan’s multiple range test, *p* < 0.05).

We assayed the K^+^ and Na^+^ contents in shoots and roots of seedlings under salinity, since the degree of stress depends on their uptake and translocation. Under control condition, shoot K^+^ was significantly lower in “YNU31-2-4” than in its parent (Figure 5A), whereas under salt stress, it was 1.4× the WT level in shoot and 2.6× in root (Figure 5A,D). Under control condition, Na^+^ levels in leaves (Figure 5B) and roots (Figure 5E) of all genotypes remained similarly low. Salinity stress increased Na^+^ concentration in WT shoots relative to the other two genotypes, reaching 6.5× that in “YNU31-2-4” plants (Figure 5B). Under control conditions, the Na^+^/K^+^ ratio did not differ significantly among the tested genotypes in shoots (Figure 5C) and roots (Figure 5F). Under salinity, it was 9.2× the “YNU31-2-4” level in WT shoots and 2.9× in WT roots (Figure 5C,F).

**Figure 6:**
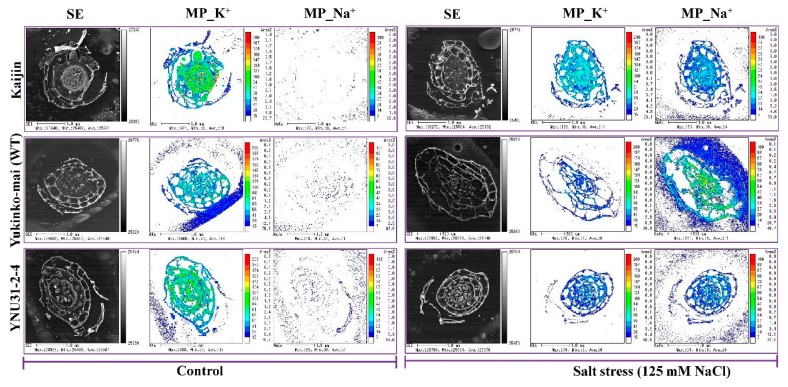
Accumulation and distribution of K^+^ and Na^+^ in cell clusters of 20-day-old seedlings. Relative amounts of K^+^ and Na^+^ are indicated by color coding. SE, secondary electron image; MP_K^+^, mapping pattern of K^+^; MP_Na^+^, mapping pattern of Na^+^.

Under the control condition, electron probe microanalysis revealed a dense distribution of K^+^ in the basal portion of the shoot of all genotypes, but only a very sparse distribution of Na^+^ (Figure 6). Under salt stress, the Na^+^ distribution in cells was increased in all genotypes, and the salt-sensitive WT accumulated significantly more Na^+^ than the other two genotypes (Figure 6).

### 2.7. Salinity Tolerance and Yield Assessment at Reproductive Stage

**Figure 7:**
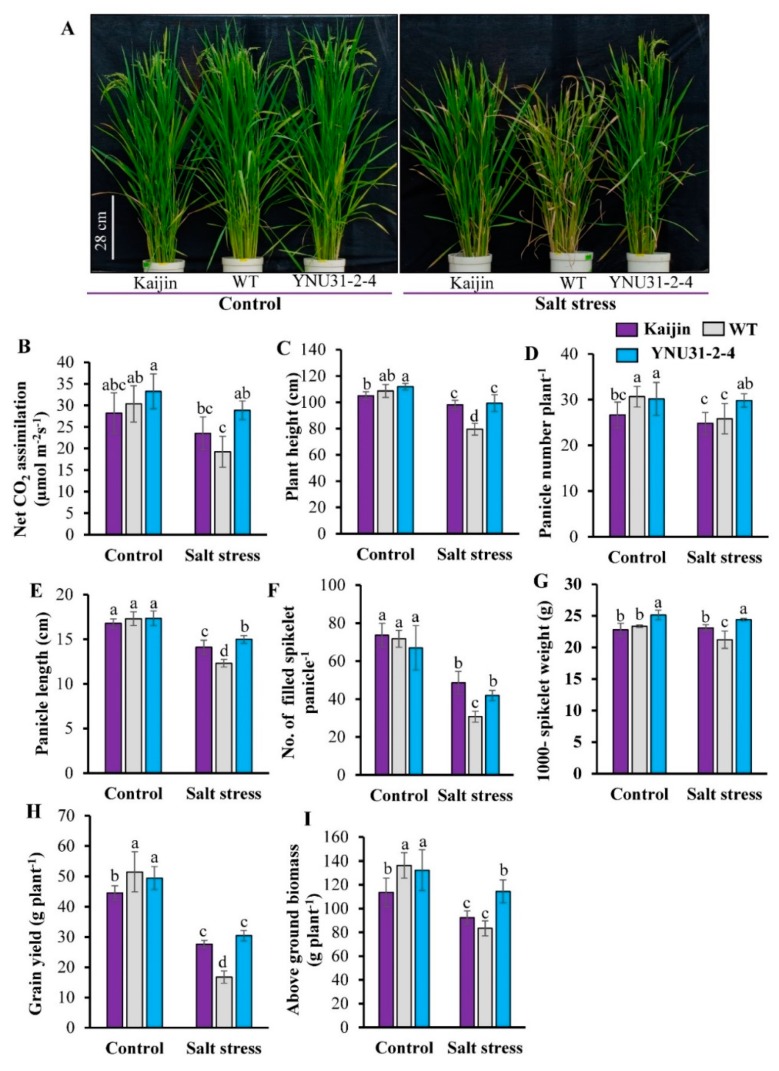
Salinity tolerance of BC_3_F_3_ line “YNU31-2-4” at heading. (**A**) Phenotypic comparison of “Kaijin”, WT, and “YNU31-2-4” plants grown with or without salt stress. Salt-stressed plants were grown in 50 mM NaCl from 60 days after germination (DAG) and then in 75 mM from 74 DAG until 95 DAG (booting stage), and then in fresh water until 110 DAG (heading). (**B**) Net CO_2_ assimilation rate of the penultimate leaf four weeks after imposition of salt treatment. (**C**) Comparison of plant height at 110 DAG. (**D–I**) Comparisons of (**D**) number of panicles/plant, (**E**) panicle length, (**F**) number of filled spikelets/panicle, (**G**) weight of 1000 filled spikelets, (**H**) grain yield/plant, and (**I**) dry weight of aboveground biomass at harvest. Data are mean ± SD of 6 individuals. Bars with the same letter are not statistically different (Duncan’s multiple range test, *p* < 0.05).

Under control condition at the reproductive stage, there was no obvious phenotypic difference between WT and “YNU31-2-4” plants (Figure 7A). Salt stress for five weeks caused severe burning and wilting symptoms in WT, but “YNU31-2-4” and “Kaijin” plants maintained green leaves. Under control condition, the penultimate leaves of “YNU31-2-4” plants maintained a slightly higher net CO_2_ assimilation rate than the parents (Figure 7B). Salt stress for four weeks significantly reduced the net CO_2_ assimilation rate of WT plants relative to “YNU31-2-4” and “Kaijin”. Under the control condition, WT and “YNU31-2-4” plants had similar phenotypic characters and yield attributes except for a higher 1000-spikelet weight than the parents (Figure 7C–G). Under salt stress, in contrast, “YNU31-2-4” had higher plant height, yield characters, and aboveground biomass than WT (Figure 7C–G,I). Relative to control condition, salt stress reduced grain yield by 68% in WT but by 38% in “YNU31-2-4” (Figure 7H). As a result, the grain yield of “YNU31-2-4” was 10% higher than that of the donor parent “Kaijin” under control condition and 45% higher than that of WT under saline condition.

## 3. Discussion

Soil salinity is a major threat to the future food production, affecting more than 6% of the total land area [54]. The rapid global warming and sea level rise pose threats to rice yield and quality in South Asian rice-growing countries. In addition, a tsunami contaminated paddy field in Miyagi prefecture, Japan, with salt in 2011 [32,37,55]. Therefore, it is important to introgress genes/QTLs/SNPs conferring salt tolerance in locally grown popular rice cultivars, focusing on higher grain yield, to ensure food security under changing climatic conditions. Cultivar improvement through conventional breeding is feasible, but it takes a long time to minimize linkage drag through phenotypic screening [56,57]. For these reasons and to achieve breeding goals, we introgressed the *hst1* gene from “Kaijin” into “Yukinko-mai”, which has excellent yield stability. We developed the BC_3_F_3_ generation, named “YNU31-2-4”, through SNP marker-assisted selection (Figure 1A).

To accelerate the breeding cycle, we used a biotron speed-breeding system (Figure S1) without a CO_2_ supply, since the application of 475 ppm CO_2_ in growth chambers did not greatly change the breeding cycle [45], and many rice breeders do not have CO_2_ regulation facilities owing to the high cost. By using a longer daylength (14/10 h light/dark) for first 30 days to accelerate the vegetative growth followed by a shorter daylength (10/14 h light/dark) to induce reproduction, tiller removal, and embryo rescue to decrease the period before seed maturity (Figure S1), we were able to achieve four breeding generations within 11 months (Table S1). Developing four to five generations a year is the ultimate objective [46,47,48]. This simplified, faster, efficient method for reducing the duration and number of breeding cycles will contribute significantly to genomic studies and the deployment of superior rice.

We used whole-genome sequencing (WGS) to characterize the advanced breeding lines and revealed genome recovery rate, genotype blocks, and putative phenotypes (Figure 2 and Figure 3; Table 1 and Table 2). WGS identified 118 454 SNPs/indels as markers. As WGS provides higher resolution of genome blocks than conventional SSR marker methods [58], it can thus be used for advancing generations from parents with low genetic variation, as here. Furthermore, in subsequent selection, knowledge of these genotype blocks helped us to rapidly fix heterozygous regions into the recipient allele through the MABC selection method (Figure S2A). Our high-resolution analysis with massive numbers of SNP/indel markers not only enabled accurate genome-wide genotyping, but also highlighted potential recombination hotspots.

Functional annotation was able to predict five SNP/indels (Table 2) that may affect agronomic traits and thus phenotype in our advanced line “YNU31-2-4”. One of two non-synonymous changes in *Os09g0356200*, a putative heading-date-associated gene identified in a gene-based genome-wide association study (GWAS) [53], caused a frameshift deletion probably causing loss-of-function. BC_3_F_3_ progeny of BC_3_F_2_ plants harboring the *Os09g0356200* gene in the heterozygous state likely shows a wide range of heading date. Indeed, the field assessment results demonstrate the difference in the distribution of heading dates between “YNU31-2-4” and WT (“Yukinko-mai”) (Table 3; Figure S2B). Additionally, the heading date of the BC_3_F_3_ population was associated with the genotype of the putative heading-date-associated gene *Os09g0356200* (Table 2; Figure S2). Our finding indicates the potential value and reliability of gene-based GWAS among the Japanese rice population data set [53] in breeding by incorporating the genetic variations into those cultivars, and its practical value in predicting genes governing complex traits of agronomic phenotypes. The results of whole-genome sequencing have demonstrated the success in developing improved cultivars using our rapid breeding system. They also reveal undesirable genome regions and genes from the donor parent “Kaijin”, valuable information for estimating agronomic properties without further phenotyping study. Identifying heterozygous genes can provide mechanistic insights toward homogenizing phenotypes for ongoing breeding. It is important to note that the heterozygote disadvantage has been overcome by taking advantage of the SNP-based selection of the homozygous WT allele from the YNU31-2-4 population (Figure S2). Furthermore, other morphological traits of “YNU31-2-4” were similar to those of WT (Table 3; Figure S3A–C). The field assessment results along with the high-resolution genotyping data indicate no apparent grain yield reduction in “YNU31-2-4” in relation to the presence of the target *hst1* gene. In fact, “YNU31-2-4” increased ca. 11% yield than the donor parent “Kaijin” owing to the higher number of panicles per plant and 1000-grain weight.

Rice is very sensitive to salt stress at the seedling stage [7], and its sensitivity varies with the developmental stage [59]. To assess the practical utility of *hst1* in our introgression line, we exposed seedlings to moderate (75 mM) and high (125 mM) salt stresses. Salt tolerance at this stage is of importance in saline environments, as crop establishment is fundamentally determined during the earliest stages of development. Our findings revealed that under high salt stress, the “YNU31-2-4” plants had a significantly higher survival rate, shoot, and root biomass than WT (Figure 4A–F), which suggest strong tolerance similar to that of the donor parent. The “YNU31-2-4” plants maintained significantly higher plant growth, proline content, and plant water status under salinity, which could indicate a physiological and biochemical tolerance mechanism [54,60]. In fact, previous studies showed that salinity might reduce the fertility of the spike and the translocation of assimilates to the grain in bread wheat and rice. Physiologically, the “YNU31-2-4” maintain its fully hydrated state under saline condition, which could at least partially have rapid and large effects on cell expansion, cell division, stomatal opening, maintain normal rates of transpiration, abscisic acid (ABA) accumulation, etc. Assay of soluble proline levels is a useful way to monitor physiological status and to assess stress tolerance, since plants under salt stress accumulate this osmoprotectant against ion-dependent protein degradation [61]. Proline is accumulated in taxonomically diverse sets of plants [62], providing stress tolerance by protecting the cell membrane and maintaining osmotic balance within the cell, and also serves as an organic nitrogen reserve during stress recovery [61,63,64].

We also assessed yield traits of “YNU31-2-4” under salt stress at the early reproductive to booting stages, when salt stress reduces panicle and spikelet numbers per plant, leading to significant yield losses [65,66]. The improvement of rice grain yield under salt stress is the focus of breeding [67]. Owing to the significant increases in panicle number per plant, spikelet number per panicle, and 1000-spikelet weight, the final grain yield of “YNU31-2-4” plants was 45% higher than WT under salt stress at the reproductive stage (Figure 7D,F–H). Under control condition, there was no yield difference between “YNU31-2-4” and WT (Figure 7H). Interestingly, “YNU31-2-4” has higher yield potential than the donor parent under control condition owing to the higher number of panicles per plant and 1000-spikelet weight, comparable to the field evaluation results (Figure 7D, G). The higher number of panicles can be attributable to the WT background. The higher seed weight of “YNU31-2-4” could be due, at least partly, to the improved photosynthetic efficiency (Figure 7B) due to coordination of leaf morphological (Table 3) and physiological (Figure 7B; Figure S4) traits, which has great potential for use in breeding for higher yield. Accordingly, “YNU31-2-4” showed larger flag leaf than the donor “Kaijin”, which could play an important role to grain filling and hence determining yield potential. The superior tiller growth with higher leaf size rendered the source, sink, and flow stronger and more harmonized and consequently increased the cereal yield [68,69,70,71,72]. Thus, this study clearly shows that the introgression of *hst1* to the WT significantly increased salt resistance without any reduction in grain yield. Thus, “YNU31-2-4” has significant breeding value without a noticeable yield penalty under normal and salt stress conditions.

Roots absorb minerals and water from the soil and play a key role in transporting them to leaves. In the context of salt tolerance, roots are sensitive to NaCl and are the first site of defense, directly limiting or excluding sodium uptake [54,73]. Roots are often used as a biomarker of salt stress. Root architecture differed between WT and “YNU31-2-4” plants after two weeks of normal hydroponic culture (Figure S5). Roots of “YNU31-2-4” and “Kaijin” exposed to high salt stress elongated more than WT roots (Figure 4D). The better morphophysiological and biochemical characters of “YNU31-2-4” under salt stress demonstrate the success of introgression of *hst1* into “Yukinko-mai”.

The genes involved in conferring salt tolerance, which is likely a complex trait controlled by a combination of multiple genes, are yet to be elucidated. Recent research advances have identified major genes conferring salinity tolerance in rice, including *OsHKT1;1*, *OsHKT2;1*, *OsSOS1*, *OsNHX1*, *OsCAX1*, *OsAKT1*, *OsKCO1*, *OsNRT1;2*, *OsCLC1*, *OsADS31* and *OsTPC1*; however, their functional pathways during salt stress are not coordinately linked for explaining the very complex phenomenon of salt tolerance [16,74,75]. The *hst1* (loss-of-function in *OsRR22*) gene primarily led to the upregulation of *OsHKT1;1* (encoding a high-affinity K^+^ transporter) that functions as a Na^+^ transporter contributing salt resistance of the *hst1* mutant and “Kaijin” [32]. In our experiment, Na^+^ content and Na^+^/K^+^ ratio in leaf and root were divergent between WT and “YNU31-2-4” and therefore may represent the effects of “basic” strategies related to salt tolerance or susceptibility. The quantification and localization results demonstrate that like “Kaijin”, “YNU31-2-4” plants maintained a very low Na^+^/K^+^ ratio in both shoot and root under salt stress (Figure 5C,F), which is one of the most important mechanisms used by plants to withstand salt stress [76,77]. Under salt stress, the susceptible WT plants had more Na^+^ densely localized in shoot tissue (Figure 6). An overload of Na^+^ can dramatically depolarize the plasma membrane, leading to K^+^ efflux via depolarization-activated outward-rectifying K^+^ channels [78]. It is notable that *hst1*-regulated salt stress resistance involved K^+^ homeostasis. These results suggest that the accumulation of more K^+^ with less Na^+^ in “YNU31-2-4” plants would be mediated by a mechanism of K^+^ influx and Na^+^ efflux. The possible roles of the high-affinity K^+^ transporter *OsHKT1;1*, upregulated by *hst1*, could mediate salt stress resistance in “YNU31-2-4”. Further investigation will be needed to elucidate the molecular mechanisms mediating K^+^ and Na^+^ homeostasis in “YNU31-2-4”.

In summary, our results demonstrate that the modified biotron breeding system coupled with SNP MAS offers a rapid and effective way to improve single traits in rice. The precise introgression of *hst1*, combined with suitable genetic resources and phenotyping results, resulted in the selection of a line, “YNU31-2-4”, adapted to salt stress at the vegetative and reproductive stages with improved yield due to improved water relations, photosynthesis, ion homeostasis, regulation of Na^+^ uptake, and xylem loading of Na^+^ to shoot. In order to corroborate the obtained salt stress data, the future perspective of this study is to evaluate the phenotype of the promising line under large-scale field trials. “YNU31-2-4” is a potential candidate for new rice cultivar with markedly improved salinity tolerance, which might sustain grain yield and food security in a changing climate.

## 4. Materials and Methods

### 4.1. Planting Materials

Seeds of “Yukinko-mai” (elite cultivar) and “Kaijin” (salt tolerant) were obtained from the Niigata Agricultural Research Institute’s Crop Research Center (Nagaoka city, Niigata, Japan) and the Iwate Biotechnology Research Center (Kitakami city, Iwate, Japan), respectively.

### 4.2. Speed-Breeding–Modified Controlled-Biotron Breeding Conditions

We developed advanced generations using the protocol described by Ohnishi et al. [45] with some modifications. Plants were grown in a growth chamber (CFH-415; Tomy Seiko, Tokyo, Japan) equipped with temperature, light, and humidity controls. Seeds were sterilized in 2.5% sodium hypochlorite and incubated at 30 °C in the dark for 2 days. They were then placed on seedling nursery trays and cultured. Ten-day-old seedlings were transplanted (1 per pot) into 230-mL plastic pots filled (4/5) with granulated rice nursery culture soil. Plants were grown under a long daylength (14/10 h light/dark) for 30 days to accelerate vegetative growth and then under a short daylength (10/14 h light/dark) to accelerate reproductive development. The temperature was maintained at 30/25 °C light/dark. Relative humidity was set to 70% and light intensity was set to 350 µmol m^−2^ s^−1^ (Figure S1). Each plant was restricted to the main culm by removing tillers. The flowers of the female parent were emasculated and pollinated according to Ohnishi et al. [45]. At 10 days after pollination, we rescued embryos from developing seeds and cultured them for 10 days according to the protocol. Healthy rice seedlings were then transplanted and raised to the next breeding step.

### 4.3. Developing Salt-Tolerant Line by Backcrossing “Kaijin” to “Yukinko-mai”

We performed backcrossing to develop an advanced line for salinity tolerance due to the *hst1* gene derived from “Kaijin” using the recurrent parent “Yukinko-mai” (Figure 1A). F_1_ plants were confirmed as heterozygous at the *hst1* (*OsRR22*) locus by Sanger sequencing, and were backcrossed to “Yukinko-mai” to produce BC_1_F_1_ plants. We followed the same strategy of selecting plants heterozygous at *hst1* and backcrossing to develop BC_2_F_1_ and BC_3_F_1_ generations. Selected BC_3_F_1_ heterozygous plants were self-pollinated to generate BC_3_F_2_ lines with the donor allele in the homozygous state. We sequenced the genome of BC_3_F_2_ line #31-2-4 to compare with the genomes of the parents. Self-pollinated seeds of line #31-2-4 were named “YNU31-2-4” (BC_3_F_3_ generation) and used for phenotypic evaluation.

### 4.4. Confirmation of Genotypes by Sanger Sequencing

We used a PCR primer set to amplify a 545-bp region around the selected SNP (nucleotide 1975 of the *OsRR22* locus) [32] from genomic DNA extracted from young leaves of 20-day-old plants using the CTAB method [79]. Well defined PCR product was gel-purified with a High Pure PCR Product Purification Kit (Roche Applied Science, Tokyo, Japan). Sanger sequencing was performed using a BigDye Terminator v. 3.1 Cycle Sequencing Kit (Applied Biosystems, Foster City, CA, USA) on a Prism 3130 Genetic Analyzer (Applied Biosystems). Sequence chromatogram data were visualized in FinchTV software (Geospiza, Inc., Seattle, WA, USA) to determine the genotype at the SNP position.

### 4.5. DNA Library Construction and Whole-Genome Sequencing

Total genomic DNA was extracted from leaves of “Yukinko-mai” and BC_3_F_2_ line #31-2-4 according to the protocol of Walbot and Warren [80] with some modifications. The quantity of genomic DNA was tested with a Qubit dsDNA HS Assay Kit (Thermo Fisher Scientific, Inc., Waltham, MA, USA) and the quality was tested by 0.8% agarose gel electrophoresis. The DNA was sent to Macrogen Japan Corp. (Sakyo-ku, Kyoto, Japan) for Illumina HiSeq X Ten sequencing with NGS libraries prepared by the TruSeq DNA PCR-Free Library Prep Kit (Illumina, Inc., San Diego, CA, USA). The sequence data have been deposited in the DDBJ Sequence Read Archive: DRR151851 (BC_3_F_2_) and DRR151852 (“Yukinko-mai”).

### 4.6. Read Mapping, Variant Calling, and Variant Annotation

“Kaijin” whole-genome sequencing reads (DRR021949, DRR021950, DRR021951, DRR021952) were downloaded from public databases. The raw paired-end reads from “Yukinko-mai”, BC_3_F_2_ #31-2-4, and “Kaijin” sequences were trimmed in Trimmomatic v. 0.33 software [81] with the following parameters: SLIDINGWINDOW, 8:20; TRAILING, 30; MINLEN, 70. The processed reads were mapped to the Nipponbare reference genome (IRGSP-1.0) by using the BWA-MEM v. 0.7.15 algorithm [82]. PCR duplicates in the binary alignment map (BAM) file of “Kaijin” were marked in Picard Tools v. 1.68 software (http://broadinstitute.github.io/picard/). Then indel realignment and base recalculation were done in Genome Analysis Toolkit (GATK) v. 3.6 software [83]. For multi-sample variant calling, we used GATK HaplotypeCaller in gVCF mode followed by GATK GenotypeGVCFs. We filtered out variants with missing data, multi-allelic sites, heterozygous sites in “Kaijin” and “Yukinko-mai”, low coverage depth (DP < 6), and low quality (QUAL < 20). We further filtered out heterozygous variants in BC_3_F_2_ #31-2-4 outside the range of 40%–60% allele frequency by a custom script, and then visualized the genotype map of BC_3_F_2_ #31-2-4 in the gtrellis package of R software [84]. We annotated variants in SnpEff v. 4.0e software [85] and summarized the results in the Python programming language. Sequentially, we extracted “HIGH”- and “MODERATE”-impact variants flagged by SnpEff and performed functional annotation analysis based on two agronomic data sets: data set 1, the Overview of Functionally Characterized Genes in Rice Online (OGRO) database [52]; and data set 2, the potential agronomic functional gene set selected by gene-based GWAS of the Japanese rice population [53]. One of non-synonymous variant in *Os09g0356200* was sequenced by Sanger sequencing using forward primer: 5’-cactggaggtcgaaactgct-3’ and reverse primer: 5’-tccggtcccagaaatgaagc-3’. The analyses were all based on gene annotation information and genome sequences from the Rice Annotation Project Database (RAP-DB: http://rapdb.dna.affrc.go.jp/).

### 4.7. Estimation of Genome Recovery Rate

We estimated the genome recovery rate of BC_3_F_2_ #31-2-4 by calculating the “Yukinko-mai”-type allele frequency out of total variants as:

$$\mathrm{Genome}\;\mathrm{recovery}\;\mathrm{rate}\;=\;\frac{\mathrm{YY}\;+\;\mathrm{YK}/2\;}{\mathrm{YY}+\mathrm{YK}+\mathrm{KK}}$$

where YY = number of “Yukinko-mai” homozygous variants, YK = number of heterozygous variants, and KK = number of “Kaijin” homozygous variants.

### 4.8. Phenotypic Evaluation under Field Condition

We grew “YNU31-2-4” plants in paddy fields of the Crop Research Center, Niigata University, Japan (37°51′20.75″N 138°57′37.9″E), during May-September in 2018, to evaluate the major agro-morphological traits. The experiment was laid out in a randomized complete block design with three replications. We used “Kaijin” and “Yukinko-mai” as salt tolerant and high yielding check cultivars, respectively. Thirty-day-old seedlings were transplanted at a spacing of 20 cm × 15 cm. All agronomic practices were performed uniformly for all the genotypes following the local cultural practices. Four uniform looking plants of each genotype from the central row of each replication were selected to determine the phenotype. The major agronomic traits such as (1) flag leaf color, (2) flag leaf length (3) flag leaf width, (4) heading date, (5) plant height, (6) tiller number, (7) panicle number, (8) panicle length, (9) spikelet number, (10) grain number, (11) 1000-grain weight, (12) seed setting rate, (13) grain yield, and (14) above ground biomass were determined. Grain length, width, and thickness were determined with a rice grain grader (RGQI20A; Satake, Hiroshima, Japan).

### 4.9. Growth Conditions and Evaluation of Salinity Tolerance at Seedling Stage

We evaluated “YNU31-2-4” and the parents for seedling-stage salt tolerance in the growth chamber at 26/23 °C (12/12 h) and a relative humidity of 70%. Pre-germinated seeds were placed in 230-mL plastic pots filled with rice nursery culture soil containing 0.5 g N, 0.9 g P, and 0.5 g K/kg. The experiment consisted of four treatments: 0 (control), 50, 75, and 125 mM NaCl (pH 5) The salt stress was imposed ten days after germination. The experiment used four biological replicates, each with 10 seedlings. Phenotype was evaluated 2 weeks after salt was imposed.

### 4.10. Determination of Leaf Relative Water Content, Chlorophyll, and Proline

A separate experiment was conducted to measure biochemical and physiological traits related to salinity tolerance. “YNU31-2-4”, “Kaijin”, and “Yukinko-mai” seedlings were cultured hydroponically [86]. Ten-day-old rice seedlings were subjected to 0 or 125 mM NaCl (pH 5.0). Samples were collected 10 days after salt was imposed.

The relative water content (RWC%) of control and salt-treated leaves was determined according to Sade et al. [87] as:

%RWC = (fresh weight − dry weight)/(turgid weight − dry weight) × 100

Fully expanded leaves of plants cultured in the absence or presence of salt stress were harvested at the end of the light period, snap-frozen, and ground to a fine powder in liquid nitrogen with a pestle and mortar. Total chlorophyll content was determined according to Lichtenthaler [88]. Free proline content was measured by a colorimetric assay as described by Bates et al. [89].

### 4.11. Measurement of Na^+^ and K^+^ Concentrations

Sodium and potassium ions in shoots and roots were quantified by a wet digestion method [90]. Dried, finely powered plant samples (50 mg) were digested in HNO_3_/H_2_O_2_ solution (2:1) in a microwave oven for 4–5 min until the solution became clear. The digested solution was shaken gently and filtered through 0.2-µm filters (Whatman, Maidstone, England), and the solid fraction was discarded. The contents of Na^+^ and K^+^ in the extract were quantified by atomic absorption spectrophotometry (Z-6100, Hitachi, Tokyo, Japan).

For localization of sodium and potassium ions, we prepared samples according to the protocol of Mitsui et al. [91]. Harvested basal portions of shoots were immediately frozen and embedded in OCT compound medium (Sakura Finetek USA, Inc., Torrance, CA, USA), which contained 10.24% w/w polyvinyl alcohol, 4.26% *w*/*w* polyethylene glycol, and 85.50% *w*/*w* of a nonreactive ingredient. Then 5-µm sections were scanned with an electron probe microanalyzer (EPMA-1605; Shimadzu, Kyoto, Japan).

### 4.12. Evaluation of Salt-Stress Tolerance at Reproductive Stage

“YNU31-2-4”, salt-tolerant “Kaijin”, and susceptible “Yukinko-mai” plants were evaluated for salt-stress tolerance at the reproductive stage in a semi-controlled greenhouse. Thirty-day-old seedlings were transplanted (1 per bucket) into 2.5-L plastic bucket and each treatment had six replicates. Plants were subjected to 0 or 50 mM NaCl in irrigation water (pH 5) at 60 days after germination (DAG). After 2 weeks, the salt concentration was increased to 75 mM until booting stage (95 DAG), and then plants were recovered by irrigating with fresh water. The net assimilation rate of penultimate leaves was measured with an LI-6400 gas exchange system (LI-COR Inc., Lincoln, NE, USA) 4 weeks after salt was imposed. Gas exchange was determined at 25 °C at a photosynthetic photon flux density of 350 μmol m^−2^ s^−1^. Yield and its attributes, particularly panicle number, spikelet number, and 1000-spikelet weight were determined at harvest.

### 4.13. Statistical Analysis

Values are presented as mean ± standard deviation (SD). Means were tested by analysis of variance (ANOVA) followed by Tukey’s or Duncan’s multiple range test at *p* ˂ 0.05 in SPSS software (SPSS Inc., Chicago, IL, USA).

## Figures and Tables

**Table 1:** Single nucleotide polymorphism (SNP)/indel detection in BC_3_F_2_ #31-2-4. SNPs/indels in BC_3_F_2_ #31-2-4 are classified into “Yukinko-mai”-type (homo[zygous]), “Kaijin”-type (homo(zygous)), and Hetero(zygous) alleles.

Genotype	Number of Variants			Genome %
	SNPs	Indels	Total	
‘Yukinko-mai” (homo)	84 927	21 361	106 288	89.7
“Kaijin” (homo)	2329	828	3157	2.7
Hetero	7556	1453	9009	7.6
Total	94 812	23 642	118 454	100.0

**Table 2:** List of agronomic-trait-related genes harboring alternative protein sequences in BC_3_F_2_ #31-2-4. Chr., chromosome; Ref., Nipponbare reference allele; Hetero, heterozygous. Dataset 1, Overview of Functionally Characterized Genes in Rice Online database (OGRO) [52]; Dataset 2, potential agronomic functional gene set by gene-based GWAS of Japanese rice population [53].

Chr./Position	Ref. SNP/Indel	Genotype			Alteration Type	RAP ID	Protein Encoded	Dataset 1	Dataset 2
		BC_3_F_2_	“Kaijin”	“Yukinko-mai”					
Chr06/26277010	G	A	A	G	missense SNP	Os06g0644200	Vacuolar HD-translocating inorganic pyrophosphatase 1	Cold tolerance	–
Chr08/6268486	GCACGGCCACGGC	Hetero	GCACGGCCACGGC	G	in-frame deletion	Os08g0207500	Zn-regulated transporter, iron (Fe)-regulated transporter–like protein 4	Other soil stress tolerance	–
Chr08/26913261	G	Hetero	G	A	missense SNP	Os08g0538300	Chitin elicitor receptor kinase 1	Blast resistance	–
Chr09/11449688	G	Hetero	G	A	missense SNP	Os09g0356200	Malectin-like carbohydrate-binding domain–containing protein	–	Days to heading
Chr09/11449800	AGG	Hetero	AGG	AC	frameshift deletion			–	

**Table 3:** Growth and yield performance of “Kaijin”, WT, and “YNU31-2-4” genotypes under normal field conditions. Grain yield is weight of filled spikelets at 14% moisture content. Data are mean ± SD (*n* = 10–12). Values with the same letter within a row are not statistically different (Duncan’s multiple range test, *p* < 0.05).

Agronomic Traits	Genotype		
	“Kaijin”	WT	“YNU31-2-4”
Flag leaf greenness (SPAD value)	42.93 ± 2.63 a	43.49 ± 1.82 a	43.24 ± 2.42 a
Flag leaf length (cm)	27.21 ± 2.53 b	29.85 ± 2.13 a	29.12 ± 1.85 a
Flag leaf width (cm)	1.11 ± 0.06 b	1.23 ± 0.10 a	1.21 ± 0.04 a
Days-to-heading (day)	105.17 ± 1.40 a	105.08 ± 1.38 a	103.75 ± 1.76 b
Plant height (cm)	98.42 ± 3.56 a	94.75 ± 3.49 b	95.83 ± 2.41 ab
Tiller number per plant	20.40 ± 3.31 b	23.80 ± 3.22 a	24.40 ± 3.57 a
Panicle number per plant	20.40 ± 3.31 b	23.80 ± 3.22 a	24.20 ± 3.12 a
Panicle length (cm)	18.71 ± 0.69 b	19.79 ± 0.52 a	19.11 ± 0.97 ab
Spikelet number per panicle	71.99 ± 8.91 a	73.55 ± 6.27 a	72.66 ± 5.80 a
Grain number per panicle	63.61 ± 8.20 a	67.36 ± 5.81 a	60.59 ± 7.29 a
1000-grain weight (g)	22.42 ± 0.14 b	21.56 ± 0.22 c	23.29 ± 0.43 a
Seed setting rate (%)	88.34 ± 3.71 a	91.61 ± 2.52 a	83.43 ± 7.58 b
Grain yield (g per plant)	33.77 ± 4.24 b	40.07 ± 4.59 a	37.84 ± 4.46 a
Aboveground biomass (g per plant)	61.80 ± 8.50 b	72.30 ± 7.63 a	67.13 ± 9.14 ab
Grain length (mm)	5.05 ± 0.03 b	5.17 ± 0.01 a	5.21 ± 0.06 a
Grain width (mm)	2.78 ± 0.01 b	2.66 ± 0.01 c	2.86 ± 0.04 a
Grain thickness (mm)	2.14 ± 0.01 a	2.07 ± 0.01 c	2.10 ± 0.01 b

## Supplementary Materials

The following are available online at https://www.mdpi.com/1422-0067/20/10/2585/s1. Table S1: Cross-efficiency rate and advanced generation duration in biotron breeding system. Table S2: Annotation of SNPs and indels in BC_3_F_2_ #31-2-4. Figure S1: Schematic representation of biotron speed-breeding system. Figure S2: Frameshift deletion in *Os09g0356200* affects days-to-heading of YNU31-2-4 plants. Figure S3: Agronomic trait assessment of “Kaijin”, WT, and “YNU31-2-4” plants under control field condition. Figure S4: “YNU31-2-4” maintains higher relative water, chlorophyll, and proline contents at the seedling stage under salt stress. Figure S5: “YNU31-2-4” plants have an enlarged root system.
