# Supplementary material for: Salt Tolerance Improvement in Rice through Efficient SNP Marker-Assisted Selection Coupled with Speed-Breeding
Source: Int J Mol Sci. 2019 May 26;20(10):2585. doi: 10.3390/ijms20102585 (PMC6567206; doi:10.3390/ijms20102585)

Supplementary material

Supplementary Table 1: Cross-efficiency rate and advanced generation duration in biotron breeding system. Data are mean ± SD (*n* = 5-13). Days to flowering refers to the flowering of 'Yukinko-mai' (1st breeding event), F_1, BC_1F_1, BC_2F_1, BC_3F_1, and BC_3F_2 individuals. Data in parentheses in columns 2 and 3 represent the total numbers. †Embryo rescue and tiller removal were not performed so as to obtain the maximum number of true seeds.

Supplementary Table 2: Annotation of SNPs and indels in BC_3F_2 #31-2-4.

Supplementary Figure 1: Schematic representation of speed-breeding technique based on a biotron that controls temperature, daylength, and humidity, tiller removal, and embryo rescue.

Supplementary Figure 2: Frameshift deletion in *Os09g0356200* affects days-to-heading of 'YNU31-2-4' plants. (**A**) Frameshift deletion in *Os09g0356200*; red box indicates indel position (Chr. 9:1144980). (**B**) Distribution of days-to-heading. Genotypes: Y, 'Yukinko-mai' homozygous; H, heterozygous; K, 'Kaijin' homozygous. 'YNU31-2-4' plants were grown under normal field conditions to assess differences in heading within the population.

Supplementary Figure 3: Agronomic trait assessment of 'Kaijin', WT, and 'YNU31-2-4' plants under control field condition. (**A**) Phenotypes of plants after flag leaf emergence (95 days after germination); (**B**) mature rice seeds and (**C**) brown rice grains.

Supplementary Figure 4: 'YNU31-2-4' maintains higher relative water, chlorophyll, and proline contents at the seedling stage under salt stress. (**A**) Relative water content, (**B**) total chlorophyll content, and (**C**) proline content in shoots of 20-day-old seedlings. Data are mean ± SD of three independent biological replicates. Bars labeled with the same letter are not statistically different (Duncan's multiple range *test*, $P < 0.05$).

Supplementary Figure 5: 'YNU31-2-4' plants have an enlarged root system. (**A**) Root phenotype comparison of 'Kaijin', WT, and 'YNU31-2-4'. Seedlings were cultured in Yoshida's nutrient solution and photographed at 2 weeks. (**B**) Root length and (**C**) root fresh weight of plants grown under stress-free condition for 2 weeks. Data are mean ± SD of four independent biological replicates, each being a pool of 5 individuals. Bars labeled with the same letter are not statistically different (Duncan's multiple range test, $P < 0.05$).

Supplementary Table 1. Cross-efficiency rate and advanced generation duration in biotron breeding system.

Breeding event	No. of spikelets crossed	No. of seed developed	Success rate (%)	Days to flowering	Total duration (days)
‘Kaijin’ × ‘Yukinko-mai’	9.00 ± 3.24 (81)	4.66 ± 2.44 (42)	53.84 ± 25.83	67.0 ± 1.58	77.4 ± 1.14
F_1 × ‘Yukinko-mai’	8.38 ± 2.33 (67)	5.00 ± 2.45 (40)	57.82 ± 13.37	69.4 ± 1.14	79.4 ± 1.14
BC_1F_1 × ‘Yukinko-mai’	9.15 ± 3.02 (119)	6.15 ± 2.12 (80)	68.85 ± 16.22	70.4 ± 1.67	80.6 ± 1.51
BC_2F_1 × ‘Yukinko-mai’	9.41 ± 2.31 (113)	6.25 ± 2.70 (75)	64.95 ± 14.37	70.0 ± 1.41	80.0 ± 1.41
BC_3F_1-selfing	–	–	–	71.2 ± 1.30	81.2 ± 1.30
BC_3F_2-selfing	–	–	–	70.6 ± 1.67	107.8 ± 4.15†

Supplementary Table 2: Annotation of SNPs and Indels in BC3F2 (#31-2-4)

Location/Variant type	Yukinko-mai (Homo)		Kaijin (Homo)		Heterozygous	
	SNPs	Indels	SNPs	Indels	SNPs	Indels
1kb DOWNSTREAM	10,715	3,547	216	113	666	204
3'UTR	2,877	882	86	26	180	51
INTRON_others	11,570	3,902	438	215	1335	325
INTRON_Splice donor	7	7	-	-	2	1
INTRON_Splice acceptor	10	7	-	1	1	1
CDS_Splice region	272	51	18	9	16	5
CDS_Synonymous	2,856	141	75	3	262	2
CDS_Nonsynonymous	2,839	16	70	1	230	-
CDS_Inframe INDEL	-	285	-	10	-	7
CDS_Frameshift	-	277	-	20	-	9
5'UTR	1,629	493	23	12	75	24
1kb UPSTREAM	11,735	3,640	255	111	914	289
INTERGENIC	68,341	16,751	1,807	611	5,984	1,143

Supplementary Figure 1

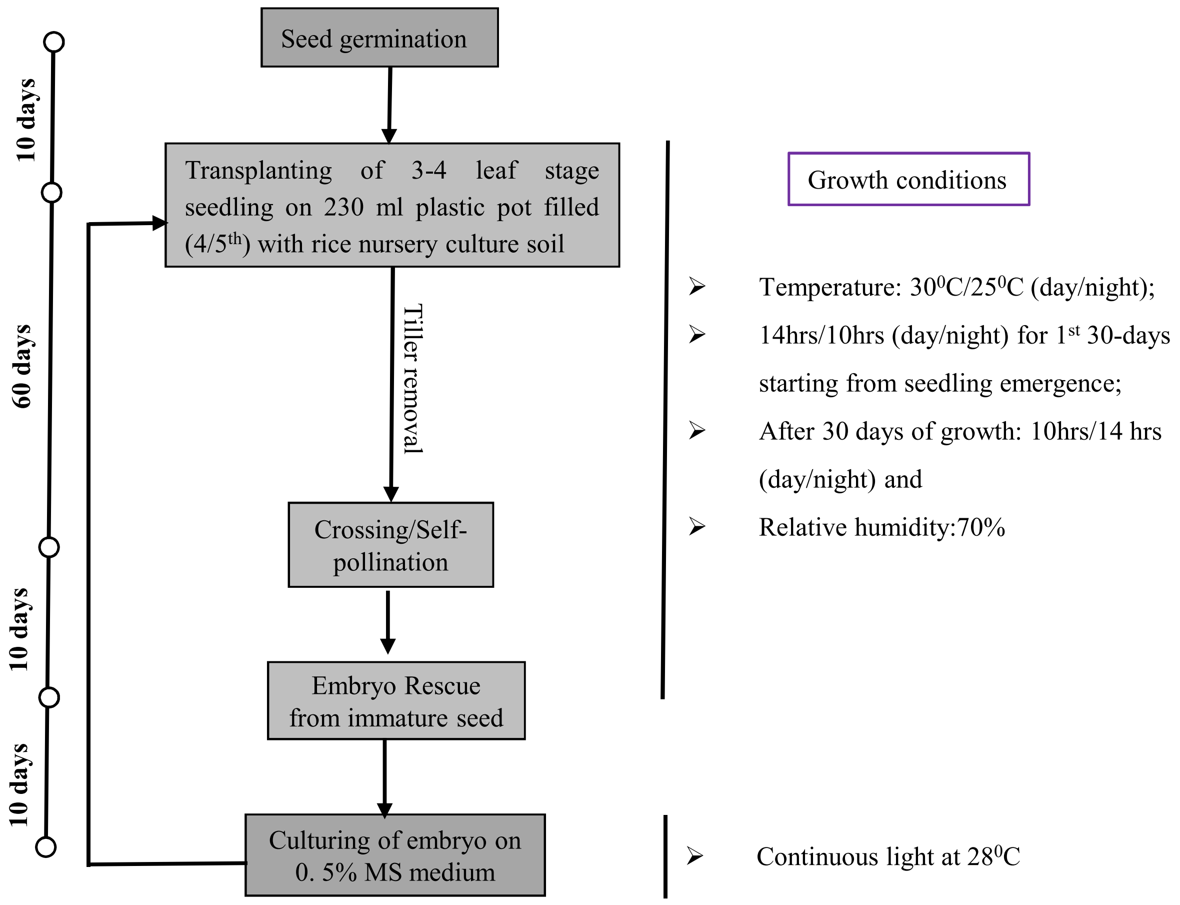

Supplementary Figure 2

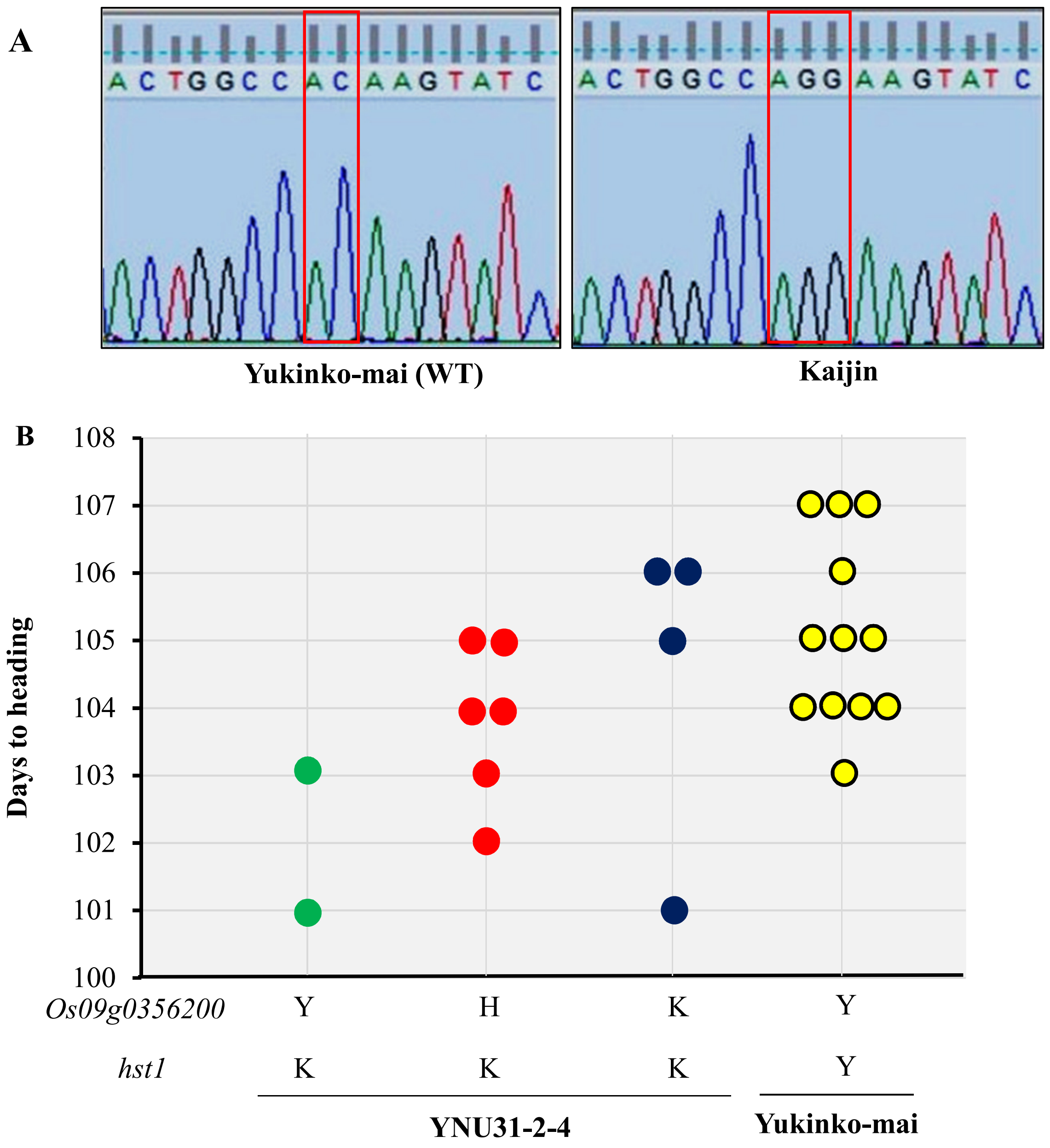

Supplementary Figure 3

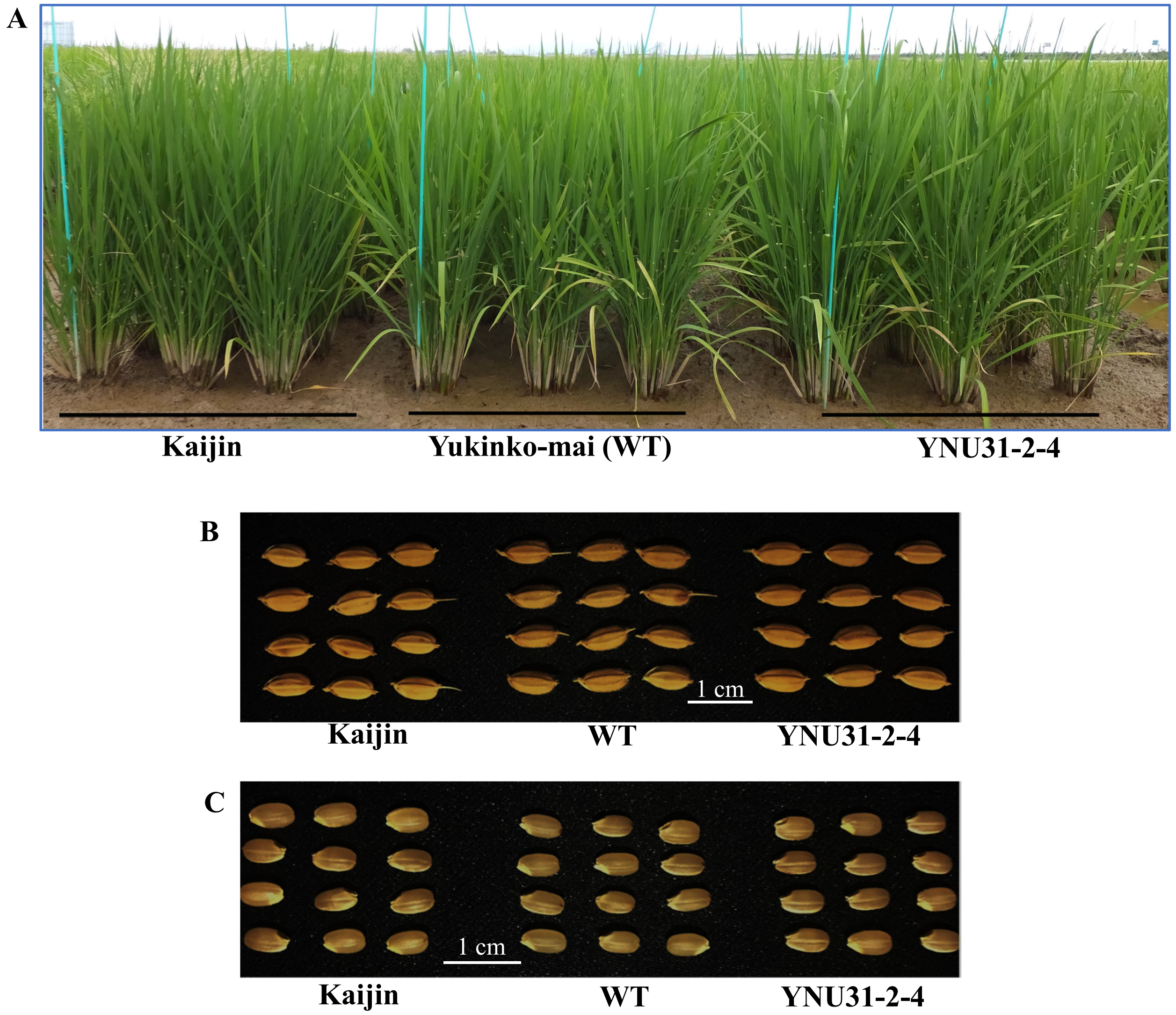

Supplementary Figure 4

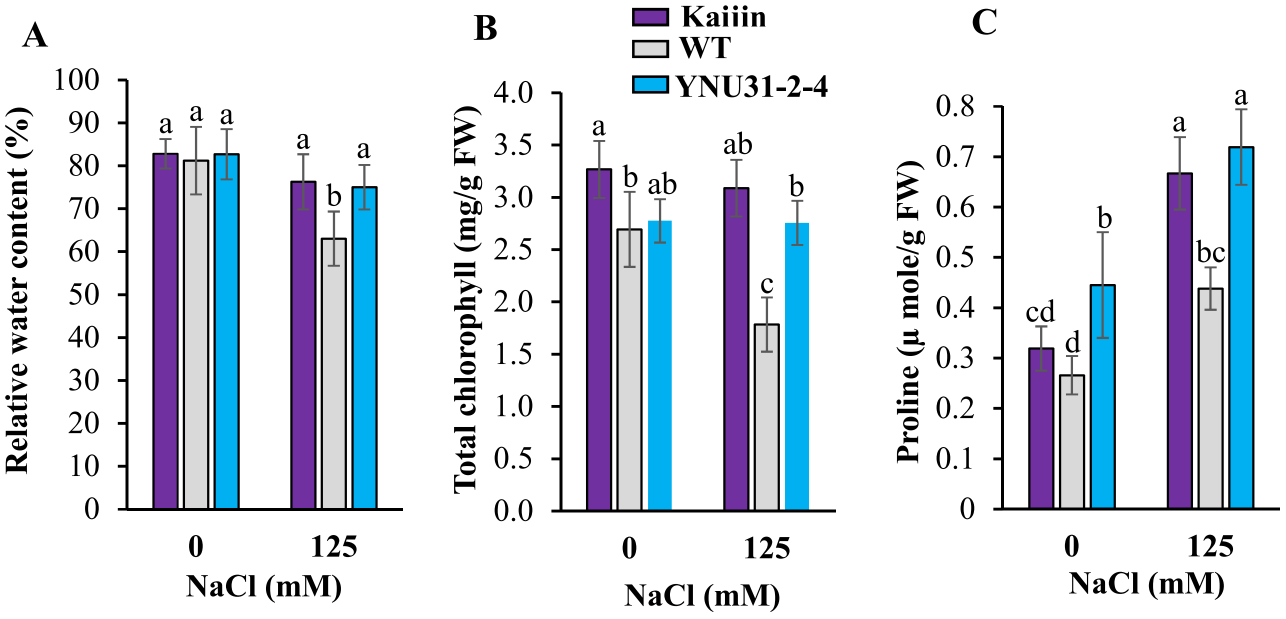

Supplementary Figure 5

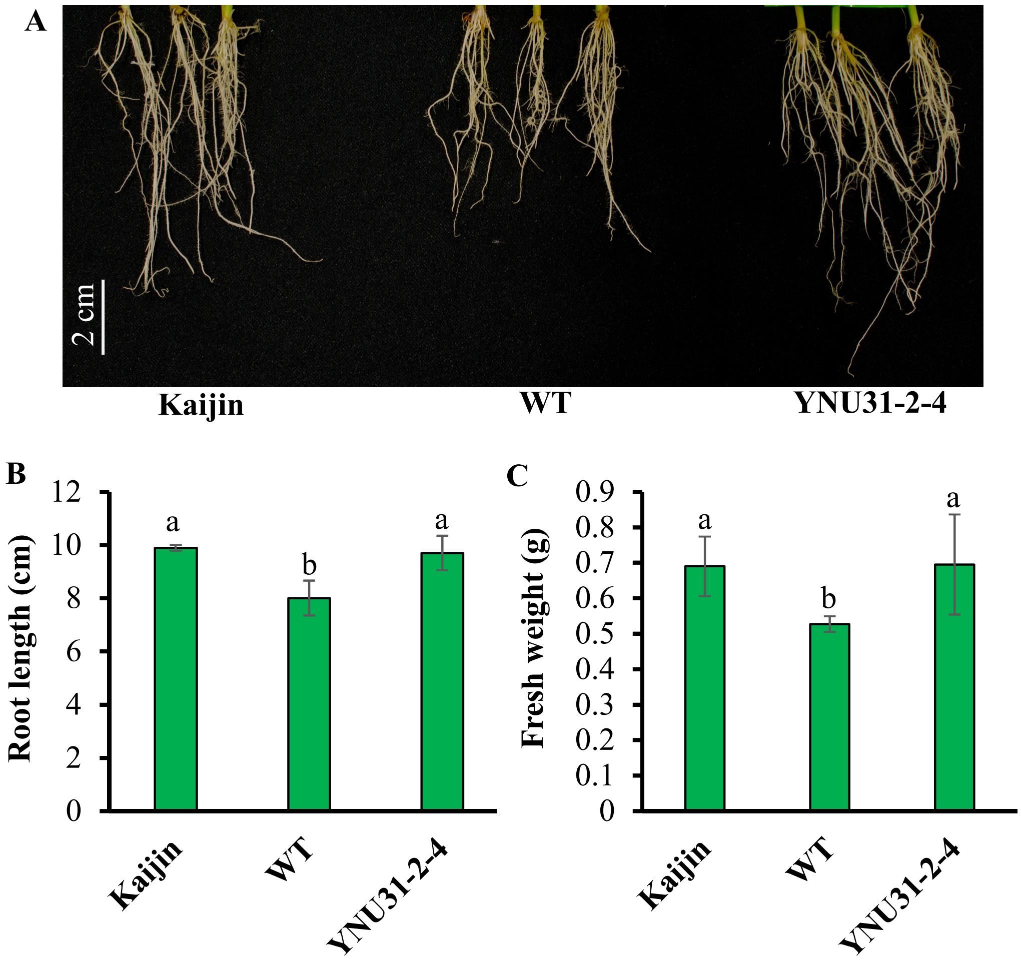

Supplement: Supplementary file 1 [file ijms-20-02585-s001.pdf]
